# The Behavioral Implications of a Multi-Individual Bonebed of a Small Theropod Dinosaur

**DOI:** 10.1371/journal.pone.0064253

**Published:** 2013-05-15

**Authors:** Lucio M. Ibiricu, Rubén D. Martínez, Gabriel A. Casal, Ignacio A. Cerda

**Affiliations:** 1 Laboratorio de Paleontología, Centro Nacional Patagónico (CONICET-CENPAT), Puerto Madryn, Chubut, Argentina; 2 Laboratorio de Paleovertebrados, Universidad Nacional de la Patagonia San Juan Bosco, Comodoro Rivadavia, Chubut, Argentina; 3 Consejo Nacional de Investigaciones Científicas y Tecnológicas (CONICET), Instituto de Investigación en Paleobiología y Geología, Universidad Nacional de Río Negro, Museo Carlos Ameghino, Belgrano, Paraje Pichi Ruca (predio Marabunta), Cipolletti, Río Negro, Argentina; Ludwig-Maximilians-Universität München, Germany

## Abstract

**Background:**

Central Patagonia, Argentina, preserves an abundant and rich fossil record. Among vertebrate fossils from the Upper Cretaceous Bajo Barreal Formation of Patagonia, five individuals of the small, non-avian theropod dinosaur *Aniksosaurus darwini* were recovered. Group behavior is an important aspect of dinosaur paleoecology, but it is not well-documented and is poorly understood among non-avian Theropoda.

**Methods/Principal Findings:**

The taphonomic association of individuals from the Bajo Barreal Formation and aspects of their bone histology suggest gregarious behavior for *Aniksosaurus*, during at least a portion of the life history of this species. Histology indicates that the specimens were juvenile to sub-adult individuals. In addition, morphological differences between individuals, particularly proportions of the appendicular bones, are probably related to body-size dimorphism rather than ontogenetic stage.

**Conclusions/Significance:**

Gregarious behaviour may have conferred a selective advantage on *Aniksosaurus* individuals, contributing to their successful exploitation of the Cretaceous paleoenvironment preserved in the Bajo Barreal Formation. The monospecific assemblage of *Aniksosaurus* specimens constitutes only the second body fossil association of small, coelurosaurian theropods in South America and adds valuable information about the paleoecologies of non-avian theropod dinosaurs, particularly in the early Late Cretaceous of Patagonia.

## Introduction

Dinosaur behavior is difficult to infer from the fossil record. The majority of behavioral studies are based on track sites and bone beds [1], [2]. Complex social behaviors, such as gregariousness (herding, flocking, etc.) have frequently been inferred from these kinds of data, as well as from associations of bones of conspecific individuals. For example, many ornithischian species have been interpreted as gregarious based on these types of data [2], [3], [4], [5], and many sauropod dinosaurs have been interpreted as gregarious based on trackways and associations of juvenile and adult individuals [6]. In contrast, evidence of gregariousness in Theropoda, particularly non-avian theropods, is controversial [7], [8], but similarly includes trackways [7], [9], [10], [11] as well as extrapolation from the behaviors of extant theropods (birds). However, among non-avian theropods, associations of multiple conspecific individuals are less common than in other groups of dinosaurs. Despite frequently made assumptions, such accumulations of conspecific individuals are not always a consequence of the presence of gregariousness [7], [8].

**Figure 1:**
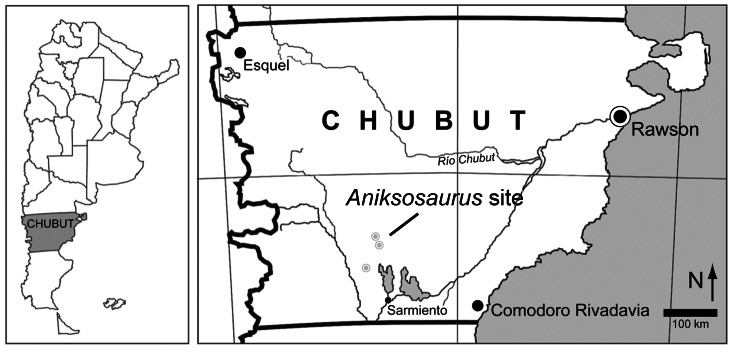
Geographic location of the *Aniksosaurus darwini* quarry (modified from [17]).

*Aniksosaurus darwini*, the small theropod examined here, was recovered from the Upper Cretaceous (Cenomanian–Turonian) Bajo Barreal Formation of central Patagonia, Argentina [12] (Figure 1). This theropod displays several morphological features that support its assignment to Coelurosauria, probably as a basal member of the group [12] (see below). Materials thus far referred to this taxon include the holotype and associated partial skeletons of in total five individuals recovered from a single 4 m by 2 m quarry. Martinez and Novas [12] suggested that fusion of the parts of the axial elements (i.e., neural arches to centra, with the exception of one indeterminate centrum on which the synostosis is well marked) indicates that at least some of the individuals are adults. Nevertheless, numerous long bones recovered from the quarry exhibit noticeable differences in proportions and other features. On the basis of taphonomic association, they suggested that this grouping of conspecific coelurosaurians was an example of gregarious behavior in this non-avian theropod taxon.

We tested the hypothesis of gregarious behavior in *Aniksosaurus darwini* using taphonomic, morphological, and histological approaches. We attempted to elucidate: 1) whether the assemblage is truly monospecific based on morphologic data; and, 2) if this is the case, whether or not all the individuals died at the same time based on taphonomic data. The results were analyzed in a paleobiological framework, particularly addressing the question as to whether the morphological differences in *Aniksosaurus* individuals provide evidence of either sexual dimorphism or ontogeny.

## Materials and Methods

**Figure 2:**
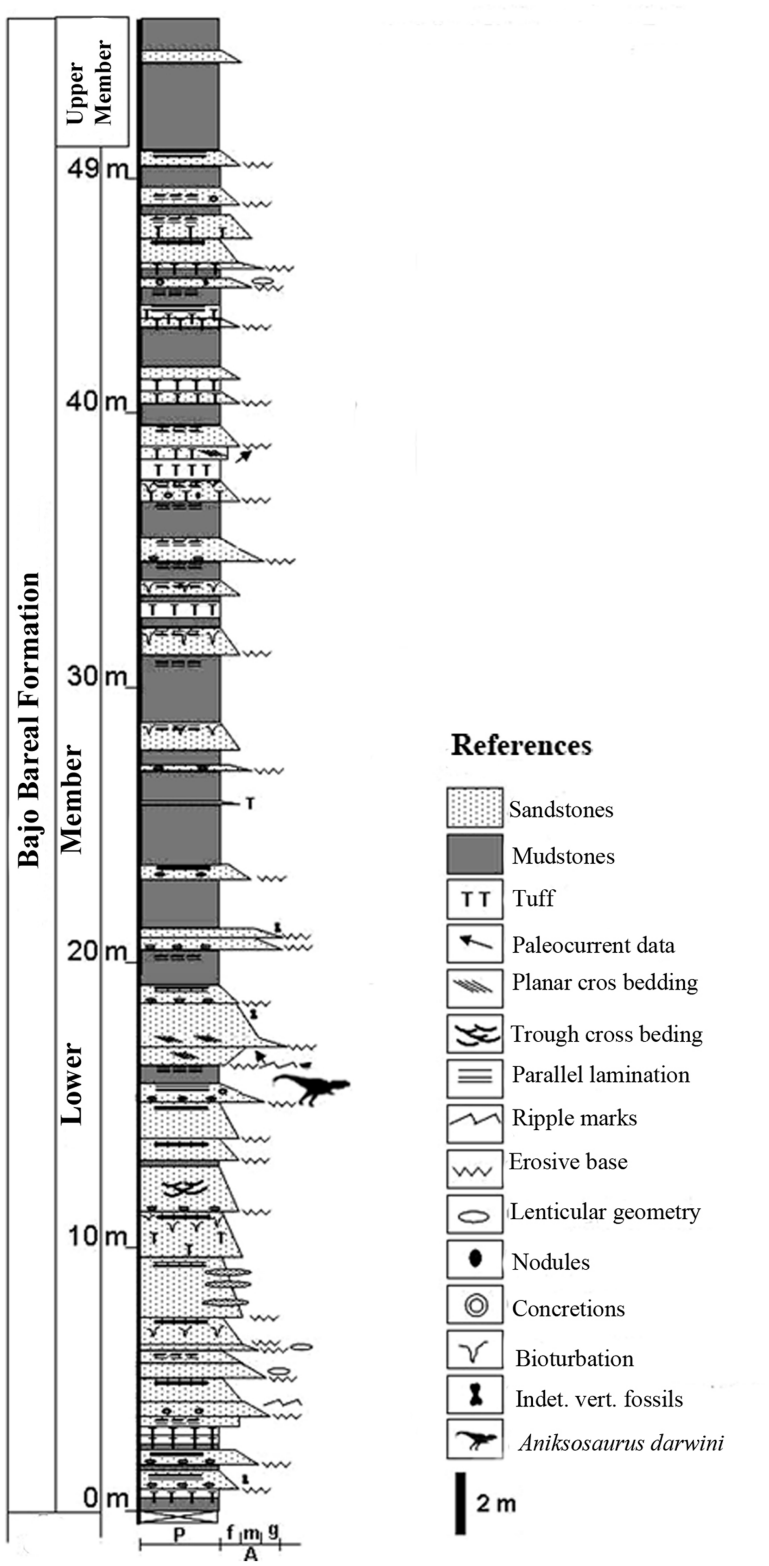
Stratigraphic column of the Bajo Barreal Formation in Chubut Province, Argentina showing the stratigraphic level at which *Aniksosaurus darwini* specimens were collected.

All specimens come from the Lower Member of the Bajo Barreal Formation, Chubut Group, Golfo San Jorge Basin. The specimens were embedded in a fining-upward, green sandstone, with an unconformable lower contact. The age of the locality is early Late Cretaceous (Cenomanian–Turonian [13]; Figure 2).

**Figure 3:**
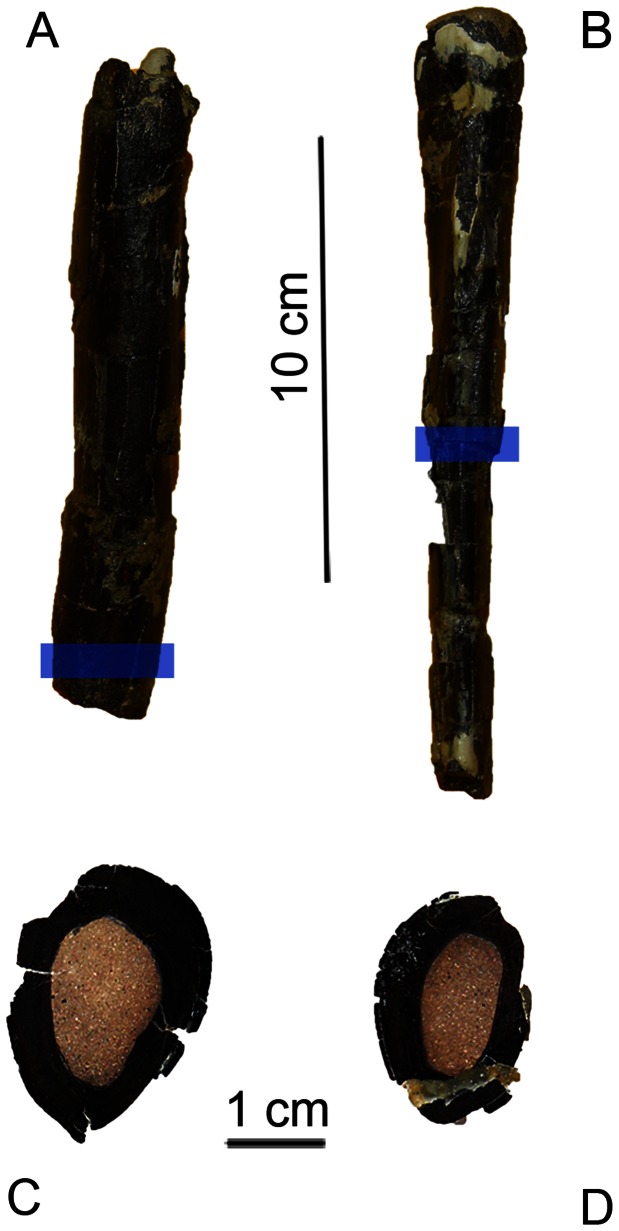
Thin sections of tibia of *Aniksosaurus darwini*. Tibiae MDT-PV 1/28 (A, C) MDT-PV 1/1 (B, D). Blue bars indicate the relative position at which the thin sections were taken.

The holotype and hypodigm of *Aniksosaurus darwini* comprises elements of the axial and appendicular skeletons of five individuals. The associated materials collected at the site include fourteen vertebrae (cervicals, dorsals, and caudals), five humeri, one fibula, five femora, seven tibiae, five pelvic bones, three phalanges, and eight indeterminate fragments (but correlated to the rest of the identifiable materials), all of *Aniksosaurus*. Herein we focus on the tibiae and femora: an articulated right hind limb, including femur and tibia (Museo Desiderio Torres, Paleovertebrados, Sarmiento, Chubut, Argentina (MDT-PV) 1/48), two left femora (MDT-PV 1/23, 1/26), two right femora (MDT-PV 1/3, 1/27), three left tibiae (MDT-PV 1/1, 1/22, 1/34), and four right tibiae (MDT-PV 1/10, 1/28, 1/44). The morphologies of these elements were described by Martínez and Novas [12]; we also examined them firsthand. In order to assess the somatic ages and ontogenetic growth stages of the studied specimens, histological thin sections were made from the mid-shafts of left (MDT-PV 1/1) and right MDT-PV 1/28) tibiae (Figure 3A–D). These two specimens were chosen on the basis of their different sizes (see below). Thin sections were prepared using the method outlined by Chinsamy and Raath [14] and studied using a petrographic polarizing microscope. Nomenclature and definitions of structures used in this study are derived from Francillont-Viellont et al. [15] and Chinsamy-Turan [16]. Lines of arrested growth (LAGs) were recognized as concentric and uninterrupted lines of discontinuity in the bone [15], [16], [17], [18]. With some uncertainty, the canals within the bone were considered reflective of the extent and organization of vascularization [19].

## Results

### Sedimentology/Paleoenvironment

The lithology and structure of the Bajo Barreal Formation at the *Aniksosaurus* site (fine, quartzose sandstone with clay intraclasts in a horizon with an unconformable base and parallel lamination: Figure 2), supports deposition by a unidirectional, low-energy, overbank fluvial deposit, specifically a floodplain deposit produced by the lateral migrating fluvial channel. A fluvial environment is additionally indicated by the superposition of sandstone tabular bodies with coarse sandstones and tuff interclasts. Paleoenvironmental studies in the Bajo Barreal Formation suggest that the sandstone deposits represent multi-episodic, cross-bedded fluvial channels [20].

### Morphologies of the Femora and Tibiae

**Figure 4:**
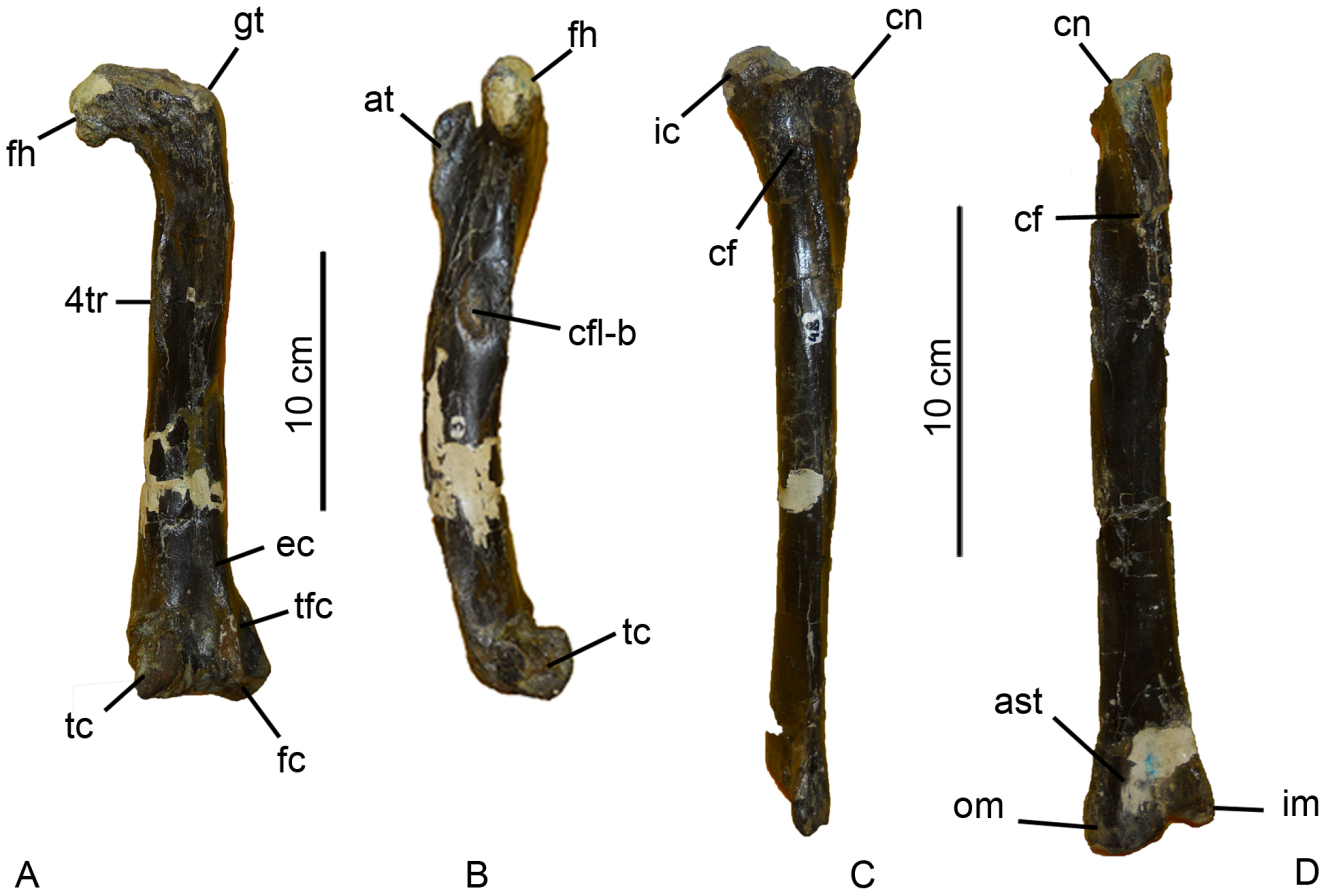
Femur and tibia of *Aniksosaurus darwini*. Right femur (MDT-PV 1/3) in posterior (A) and medial (B) views. Right tibia (MDT-PV 1/48) in lateral (C) and anterior (D) views. Abbreviations: ast, astragalar depression; at, anterior trochanter; cf, fibular crest ( = crista fibularis); cfl-bf, caudofemoralis longus/brevis fossa; cn, cnemial crest; ec, ectocondylar tuberosity; fc, fibular condyle; fh, femoral head; 4tr, fourth trochanter; gt, greater trochanter; ic, inner condyle; im, inner malleolus; om, outer malleolus; pig, posterior intercondylar groove; tc, tibial condyle; tfc, tibio-fibular crest.

We infer that the materials in general, and the long bones in particular (i.e., the femora and tibiae; Figure 4A–D), pertain to a single species for the following reasons: 1) duplicate long bones have the same morphologies; 2) the bones, consistently display theropod and coelurosaurian features; and 3) the quarry produced no evidence of any other fossil vertebrates. The femora of *Aniksosaurus* clearly differ from those of abelisaurid theropods, a group well represented in the Bajo Barreal Formation. Abelisaurid femora have proximally projecting anterior trochanters, almost at the level of the femoral head, greater trochanters that are anteroposteriorly expanded, femoral heads that are rectangular in shape, and only slightly pronounced trochanteric shelves. In contrast, in *Aniksosaurus* femora the anterior and greater trochanters lie at the same level, the fourth trochanters are reduced in size, and adductor fossae are poorly developed, all of which are characteristic of coelurosaurian theropods.

**Figure 5:**
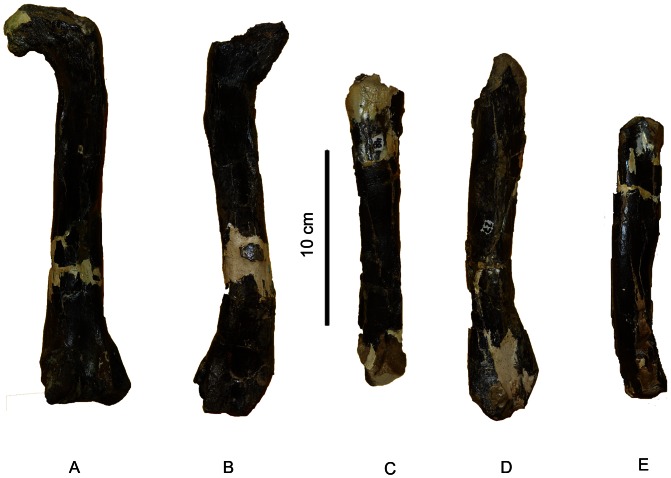
Femora of *Aniksosaurus darwini* in posterior view. (A) MDT-PV 1/3*, right femur. (B) MDT-PV 1/26*, left femur. (C) MDT-PV 1/48**, right femur. (D) MDT-PV 1/27**, right femur. (E) MDT-PV 1/23**, left femur. *Femora considered as “Femoral morph 1″ based on morphological characteristics. **Femora considered as “Femoral morph 2″ based on morpho-metrics characteristics.

The femora (Figure 4A–B), despite having abraded articular ends, are robust and relatively short compared to those of most coelurosaurians, which are gracile and elongate [12], [21] (Table 1). Although of similar anatomies, the femora can be separated into two morphs based on metrics of the shafts, fourth trochanters, and fossae for the insertion of the Mm. caudofemoralis longus and brevis (Table 1). For example, the right femur MDT-PV 1/3 and the left femur MDT 1/26 have greater shaft perimeter (taken at the distal end of the fourth trochanter), fourth trochanter length, and fossa width than MDT 1/27 (right femur), and 1/48 (right femur, holotype). Moreover, the center of the common fossa for the insertion of the Mm. caudofemoralis longus and brevis is closer to the proximal end of the femur in MDT-PV 1/26 than in MDT-PV 1/3. The femoral shaft of MDT-PV 1/48 is slightly more curved or bowed than in the rest of the femora, and MDT-PV-1/26 and 1/27 (a left and a right femur, respectively) are slightly more bowed close to the distal end than the others (Figure 5A–E).

**Table 1:** Measurements (in mm) of the femora of *Aniksosaurus darwini*.

Repository number	Tl	P	W-cflb	L-cflb	L-4tr
**MDT-PV 1/3**	250.3	105.1	20.3	34.4	61.6
**MDT-PV 1/26**	243.4*	95.2	20.1	37.1	59.2
**MDT-PV 1/48**	168.3**	92.5	20.1	33.2	51.2
**MDT-PV 1/27**	209.4	81.5	16.3	25.2	48.7

*Abbreviations: L4tr,* fourth trochanter; *L-cflb,* caudofemoralis longus-brevis fossa length; *P,* perimeter; *Tl,* total length; *W-cflF,* caudofemoralis longus-brevis fossa width.

* Articular ends partially preserved;

** Articular ends not preserved. MDT-PV 1/23 (left) is not included because it lacks the features measured.

**Figure 6:**
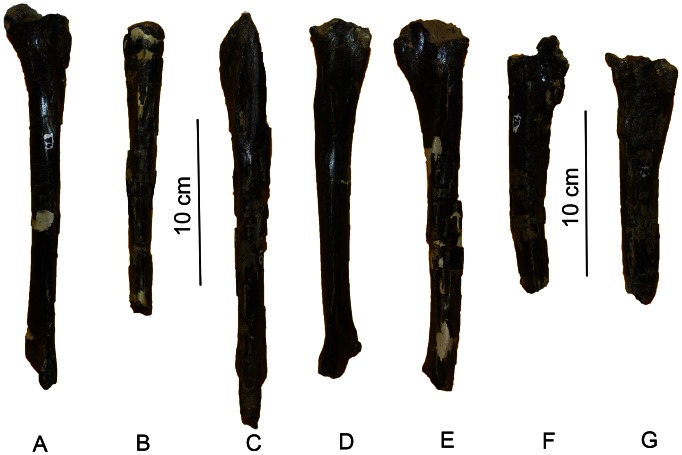
Tibiae of *Aniksosaurus darwini* in lateral view. (A) MDT-PV 1/48*, right tibia. (B) MDT-PV 1/1*, left tibia. (C) MDT-PV 1/22**, left tibia. (D) MDT-PV 1/44**, right tibia. (E) MDT-PV 1/34**, left tibia. (F) MDT-PV 1/28**, right tibia. (G) MDT-PV 1/10**, right tibia. *Tibiae considered as “Tibial morph 1″ based on morphological characteristics. **Tibiae considered as “Tibial morph 2″ based on morphological characteristics.

**Figure 7:**
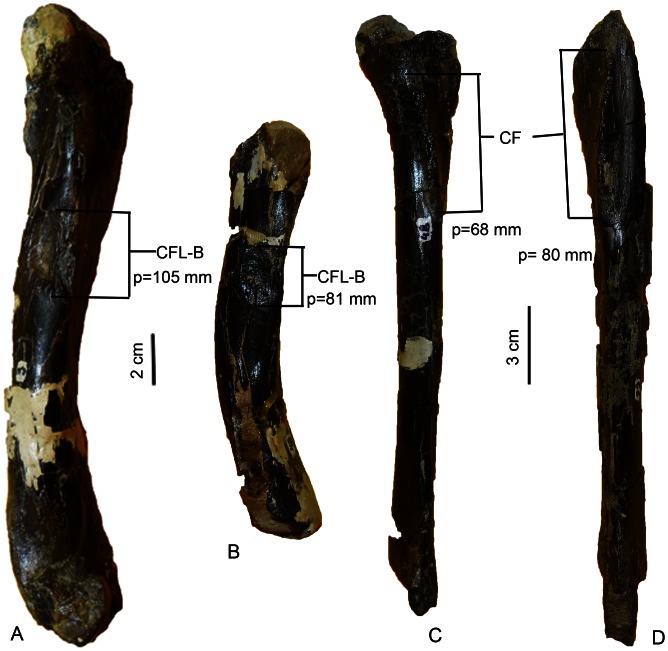
Comparisons of femora and tibiae of *Anikosaurus darwini*. (A) MDT-PV 1/3, right femur in medial view. (B) MDT-PV 1/48, right femur in medial view. (C) MDT-PV 1/28, right tibia. (D) MDT-PV 1/48, right tibia in lateral view. Anatomical abbreviations as in Figure 4 with the exception of: p, perimeter.

The long, slender, straight-shafted holotype tibia (MDT-PV 1/48), which is also the best preserved of all the tibiae, is longer than its associated femur. As with the femora, the tibiae can be segregated into two morphs (Figure 6A–G) because they exhibit different metrics, particularly in the lengths of the crista fibularis and the perimeters of the shafts (at the distal ends of the crista fibularis), as well as some morphological differences in the development of muscle scars (Table 2). Therefore, in these appendicular bones the morphological and anatomical dimensions above mentioned are considered potentially as dimorphic (see Figure 7A–D). Nevertheless, none of the individuals are interpreted as adults; therefore, these morphological characteristic could represent an ontogenetic variation, although the former hypothesis is more plausible (see below).

**Table 2:** Measurements (in mm) of the tibiae of *Aniksosaurus darwini*.

Repository number	Tl	P	L-cf
**MDT-PV 1/48**	243.1	68.5	64.5
**MDT-PV 1/22**	255.09*	80.04	80.9
**MDT-PV 1/34**	236.1*	71.1	63.6
**MDT-PV 1/44**	223.9*	71.1	55.7
**MDT-PV 1/10**	153.8*	74.4	66.5
**MDT-PV 1/28**	146.8*	80.1	60.3
**MDT-PV 1/1**	185.6*	65.5	58.01

*Abbreviations: L-cf*, crista fibularis dorso-ventral length; *P,* perimeter; *Tl*, total length. * Denote bones partially preserved.

Martinez and Novas [12], based on femoral measurements, suggested that *Aniksosaurus darwini* was approximately 2 m long and 70 cm tall at the hip, and weighed 45–65 kg. However, recent discoveries and descriptions of theropods with bones similar in proportions to *Aniksosaurus* [21], [22], [23], [24] suggested different lengths and lighter body weights. Using those comparisons, we suggest a total body length between 2–3 m and a body mass between 35–45 kg. However, the unknown total length of the tail prevents a definitive assessment of body mass for *Aniksosaurus* (see [25]).

### Bone Taphonomic Attributes

Two different taphonomic stages can be inferred in the materials. These different taphonomic attributes are directly related with increasing subaerial exposure (weathering, see [26]). The majority of the long bones exhibit the same taphonomic characteristics (eroded articular ends, moderate to intense bone surface polishing, longitudinal striation parallel to the long axis of each bone, and irregular flaking (e.g., MDT-PV 1/23; MDT-PV 1/26; MDT-PV 1/27; MDT-PV 1/1; MDT-PV 1/10; MDT-PV 1/22; MDT-PV 1/28; MDT-PV 1/34; MDT-PV 1/48, holotype femur). In contrast, MDT-PV 1/48 (holotype, tibia), MDT-PV 1/44 and MDT-PV 1/3 display slight bone surface polishing and flaking, less marked longitudinal striation and the complete preservation of the articular distal ends. These differences in taphonomic preservation and inferred differences in length of subaerial exposure of the bones, may be related with two burial stages in the overbank events (i.e., the first fluvial deposition did not cover the totality of the bones). Thus, *Aniksosaurus* specimens are considered as parautochthonous (autochthonous materials which have been reworked to some degree but not transported out of their original life habitat, see [27]). Postmortem disarticulation followed soft-tissue decomposition and the action of small, probably vertebrate, scavengers, as evidenced by the presence of small grooves on one of the femora. In contrast, neither cannibalism nor intraspecific competition is evidenced on the bones. However, theropod bite marks are in general rare, which may reflect the careful feeding strategy of those dinosaurs or that they were not habitually osteophagous [28]. Overall, the bones of *Aniksosaurus* were degraded by: 1) subaerial weathering; 2) decomposition of soft tissue; 3) scavenger action; 4) longitudinal striation caused by weathering; 5) disarticulation (with the exception of the holotype) and re-orientation by fluvial currents; and 6) differential burial in at least two stages of sedimentation.

### Bone Spatial Distribution

**Figure 8:**
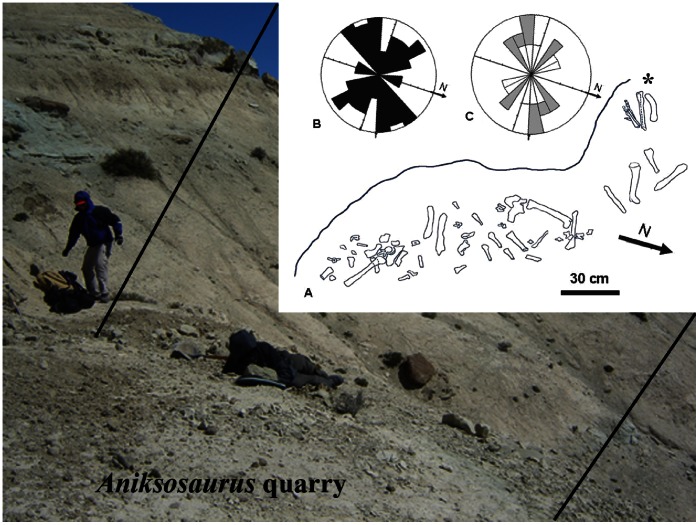
Quarry map for the *Aniksosaurus darwini* site in the Bajo Barreal Formation. (A) Map of the site, showing the distribution of the bones as recovered. An asterisk (*) denotes the location of the holotype (right hindlimb). (B) Rose diagram depicting the orientations of all bones recovered in the quarry. (C) Rose diagram depicting the orientations of only the long bones: femora (white) and tibiae (gray). The people in the photograph, RDM and M. Luna have given written informed consent, as outlined in the PLOS ONE consent form, to publication of their photograph.

As collected, the bones displayed a primary NE–SW orientation and a secondary NW–SE orientation (Figure 8). This distribution suggests transport by a unidirectional fluvial current [29]. The distribution of the long bones (Figure 8) suggests that the long bones acted as barriers for smaller elements. For example, one of the tibiae (MDT-PV 1/34) is juxtaposed next to the ilium and another tibia (MDT-PV 1/22) is placed transversely to the primary NE–SW current orientation; therefore, these long bones acted as barriers to the smaller elements. The transverse orientation of the majority of the elements with respect to stream flow, indicates that current intensity was moderate to low [30]. The combination of both disarticulated and articulated (e.g., the hindlimb of the holotype, supporting short-term distance; Figure 8) specimens implies some transport by traction and rolling on the bed of the paleo–stream channel. Transport is also suggested by abrasive rounding of the articular surfaces of the vertebrae, the total or partial absence of the articular ends and fourth trochanters of the femora, and the partial preservation of the cristae fibularis in some tibiae [31].

### Bone Diagenetic Condition

Only one bone, a caudal vertebra (MDT-PV 1/13), exhibits lithostatic compaction (permanent deformation of the rock and therefore the bones that are encompassed in it). In contrast, the remaining bones (including several tibiae and femora) exhibit transverse fracturing, transverse to the main long axis, that occurred from the weight of accumulated sediment on the bones (i.e., vertical lithostatic load). The dearth of such plastic deformation among the bones may indicate that the underlying stratum, comprising medium to fine-grained sandstones, absorbed the vertical lithostatic load of overlying unconsolidated sediment prior to mineral replacement of the bones. Based on the models of fossil assemblage proposed by Johnson [32], the accumulation of *Aniksosaurus* individuals was composed almost entirely of remains transported to the site of burial. This is supported by lateral transport (abraded eroded ends, few articulated elements, long bones showing a preferential orientation) and a long postmortem exposure. Therefore, according to Johnson [32], model III of bone accumulation is interpreted at the *Aniksosaurus* site.

### Histology

**Figure 9:**
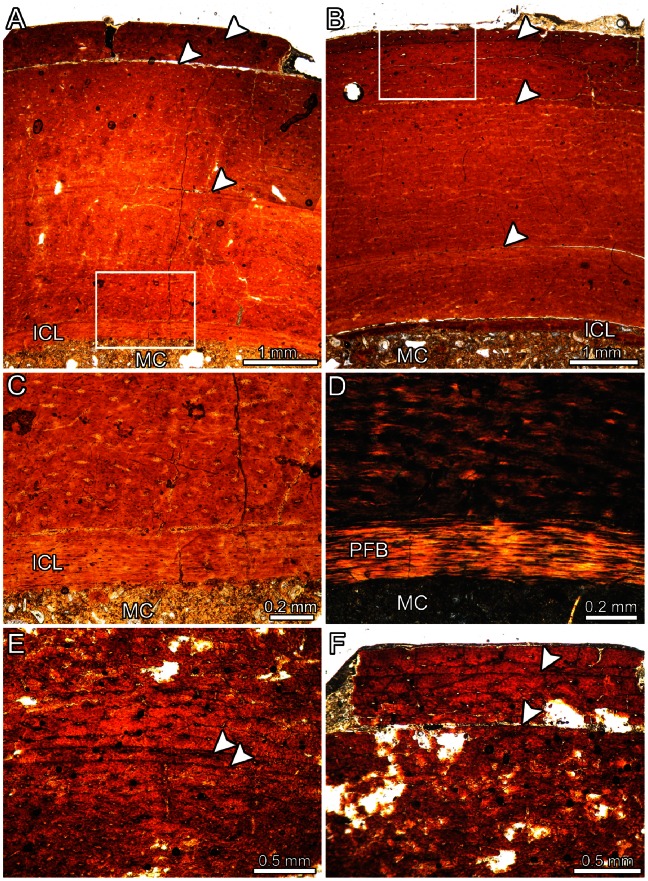
Bone histology of *Aniksosaurus darwini* tibiae. (A, B) Cyclical growth marks (arrowheads) in the cortical bone tissue of specimens MDT-PV 1/1 (A) and MDT-PV 1/28 (B). (C, D) Close-up of the inner cortex of specimen MDT-PV 1/1 (box inset in A) viewed under normal (C) and polarized (D) light. Compare the soft birefringence in some areas of the primary matrix with the strong birefringence of the ICL. (E) Double LAG (arrowheads) in the cortical tissue of specimen MDT-PV 1/1. (F) Detailed view of the outermost deposited LAGs in specimen MDT-PV 1/1. Except for (D), all figures viewed under normal light. Abbreviations: ICL, inner circumferential layer; MC, marrow cavity; PFB, parallel-fibred bone tissue.

Because the two histologically studied tibiae (MDT-PV 1/1 and 1/28) show similar microstructures, they are described together. The cortical region of the shaft is composed of compact bone and has a large marrow cavity (Figure 9A–B). The thickness of the cortical bone is almost equal in both sections (3.9–4.7 mm in MDT-PV 1/1 and 3.8–5.3 mm in MDT-PV 1/28). The perimedullary region is coated by a layer of endosteally deposited, parallel-fibred bone (the inner circumferential layer, ICL) containing flattened osteocyte lacunae (Figure 9C–D). The cortical bone tissue consists entirely of well vascularized primary bone. Vascular spaces are mainly longitudinally and circumferentially arranged, but some radial and oblique canals are also visible. Except in the outer cortex, where the vascular spaces are commonly simple and large, vascular canals are organized as primary osteons. Some vascular spaces are open to the sub-periosteal margins, and there is evidence that newly forming bone was actively depositing at the periphery. The fibrillar organization of the matrix is variable. While the intrinsic fibers of the inner cortex have a disorganized arrangement, typical of the woven-fibred bone, those located in mid- and outer cortices are well organized, showing a general anisotropy under crossed nicols. The cortical tissue is interrupted by lines of arrested growth (LAGs). The inner growth mark in both specimens consists of a double LAG (Figure 9E). The narrow (0.05–0.12 mm) spaces between these paired LAGs is composed by parallel-fibered bone in which fibers are more organized than in the surrounding, primary matrix. Two other LAGs are observed closer to the outer cortex (Figure 9F); the outermost LAG is located 0.15–0.2 mm from the sub-periosteal margin. The complete preserved zones are thick, but their thicknesses are variable (1.7–2.1 mm in the inner zone and 0.4–1.0 mm in outer zone).

## Discussion

### Behavioral Implications

The taphonomic evidence indicates that the preserved group of *Aniksosaurus* individuals died penecontemporaneously at approximately the same ontogenetic age and were transported by low to intermediate energy fluvial currents prior to burial. Therefore, the evidence supports the hypothesis that these coelurosaurian dinosaurs were living together (in aggregation) at the time of their death. The absence of evidence of cannibalism is in contrast to a similar group of *Tyrannosaurus rex* individuals [33], [34], and argues for an alternative mode of behaviour.

Based on body fossil and taphonomic evidence, several non-avian theropod taxa have been interpreted as gregarious (e.g., *Coelophysis bauri* [9], *Syntarsus rhodensis* [35], *Albertosaurus sarcophagus* [36], *Sinornithomimus dongi* [37]). In South America, body fossil evidence for gregariousness in non-avian theropods is limited to *Mapusaurus roseae* [38], [39] and probably *Bicentenaria argentina* [21] (see below). However, the South American footprint record of non-avian theropods, particularly in Chile and Peru [11], suggests gregariousness. These previous studies have pertained primarily to large theropods. This study represents the second piece of evidence (in tandem with *Bicentenaria*) in the west-central portion of Gondwana of a small coelurosaurian theropod showing some kind of group association, at least during the juvenile–subadult stage.

Communal aggregations in extant archosaurs (crocodylians and birds) and mammals are common in many taxa. These taxa engage in such behaviour at different stages and during different circumstances during life (e.g., before and after hatchling/mating, hunting packs). Varricchio et al. [37] suggested that juvenile, non-avian theropods may have herded. Our histological analysis suggests that the hypodigm collection of *Aniksosaurus* specimens pertain to juvenile–subadult individuals. Therefore, *Aniksosaurus* may have been another non-avian theropod taxon in which subadult individuals displayed gregariousness, at least during a specific interval of their life (i.e., as juvenile–subadult individuals).

The *Bicentenaria* bone assemblage, recently described by Novas et al. [21], includes bones of different-sized individuals, likely pertaining to juvenile and adult individuals [21]. This assemblage also suggests gregarious behavior for *Bicentenaria*, during at least one stage of its life. This together with our data suggests that gregarious behavior may have played an important role in the paleoecology of some basal Gondwanan coelurosaurian dinosaurs.

Roach and Brinkman [8] proposed that non-avian theropods may have been more agonistic, cannibalistic, and “diapsid-like” than bird-like in terms of sociality. Our *Aniksosaurus* data contrasts with that hypothesis and supports the inference that gregariousness (including probably some type of group sociability within a herd) could have been common in basal coelurosaurian theropods, although it is quite possible that both modes of behaviour existed. It is also possible, albeit less parsimonious, that the *Aniksosaurus* bone bed does not reflect a group aggregation but rather reflects a coincidental conspecific gathering of individuals, for example at a watering hole. This has been observed with the solitary black rhino, whose carcasses are occasionally found together by water features in the landscape [40]. Another alternative hypothesis is that the *Aniksosaurus* bone bed represents material accumulated by predators, although the lack of consistent bite marks renders this explanation unlikely.

### Ontogenetic Stage and Morphological Variation

The bone histology of the two examined specimens reveals that they were juveniles–subadults at time of death (Figure 9A–F). Evidence for immaturity includes: absence of an outer circumferential layer; absence of secondary (Haversian) osteons (even in the innermost cortex); presence of only few growth cycles; and the thickness of the zones. The growth marks (considering the double LAG as one growth mark) allowed us to infer a minimum age of three years for both sectioned specimens.

On the basis of the number, type, and distribution of growth marks, we conclude that specimens MDT-PV 1/1 and 1/28 died at approximately the same time. First, the double LAG was recorded in the cortex of the two analyzed specimens was deposited in the same growth period for both individuals (2 years prior to death). The presence of a double LAG deposited in two different specimens at the same time indicates that they probably suffered a common environmental stress during that period (assuming a similar rate of periosteal bone deposition). Hence, the studied specimens did not only died at the same ontogenetic stage, but also probably at the same time. The chances of two independent individuals in such a scenario winding up together in the same quarry, to the exclusion of any other taxa, is low. The histology, therefore, reinforces our hypothesis that the *Aniksosaurus* assemblage comprises an aggregation of juvenile–subadult individuals that lived together at the moment of death, as those seen in other groups of dinosaurs (see below, also see [5]).

As mentioned above, both the femora and tibiae display some degree of variation in some morphological features (Tables 1, 2). Two morphs are present among the appendicular bones (Figure 5A–E, Figure 6A–G). One of the morphs is characterized by more pronounced muscular and ligamentous attachments, whereas those seen in the other morphs are less marked (Figure 7A–B). Although both morphs are present among the specimens, the disarticulation of the specimens prevents a clear demonstration that those, left and right appendicular elements, characterized by more pronounced muscular and ligamentous attachments correspond to the same individual (i.e., if the left and right appendicular elements correspond one with each other and therefore; they are from the same individuals).

Interpretation of sexual dimorphism has been proposed in non-avian theropods [9], [41], [42], [43], [44], [45]. Thus, the femora of extant archosaurians (e.g., *Alligator mississippiensis*) exhibit morphological variation in their midshaft, as well as size variation in the muscular and ligamentous attachments. These morphological variations in the femora are related to sex differentiation [46]. In *Aniksosaurus,* these differences could have been related to sexual dimorphism, rather than to intraspecific variation. Nevertheless, as noted for example by Berger [40] who used extant perissodactyl death assemblages and sex ratios as a models to predict extinct group sociability, adult males and females tend to live separately (except during the mating season). Therefore, although the *Aniksosaurus* specimens are juvenile–subadults, more evidence is required in order to infer if these metrics differences seen in the tibiae and femora reflect sexual dimorphism or intraspecific variation.

## Conclusions

Based on taphonomic evidence and aspects of histology we conclude that the basal coelurosaurian theropod dinosaur *Aniksosaurus darwini* engaged in some degree of gregarious behaviour. Our histological data indicate that the individuals were juveniles–subadults. Therefore, this gregarious behaviour was manifest in at least one stage of life. In addition, the morphological size differences of the tibiae and femora could reflect sexual dimorphism rather than either ontogenetic stage or intraspecific variation, although these types of assertions are always difficult to test with the relatively sparse record of extinct dinosaurs. The precise nature of the social behaviour will probably always remain unknown; however, it is conceivable that, if indeed present in *Aniksosaurus*, that social behaviour (at least during the juvenile–subadult stage) was an advantage (e.g., in finding food, capturing prey, providing protection from other predators, and optimizing food resource) for exploiting the Cretaceous landscape represented by the Bajo Barreal Formation. The monospecific assemblage of *Aniksosaurus darwini* constitutes the second example of an aggregation of a small coelurosaurian theropod in South America. There is no doubt that non-avian dinosaurs exhibited a rich diversity of behaviours, probably every bit as complex as those exhibited by extant taxa. Our evidence sheds some light on the complexity of behaviour of non-avian theropods, hints at a role for sexual dimorphism, and augments our understanding of central Patagonian terrestrial ecosystems during the Late Cretaceous.

## References

[1] Lockley M, Schulp AS, Meyer CA, Leonardi G, Mamani DK (2002) Titanosaurid trackways from the Upper Cretaceous of Bolivia: evidence for large manus, wide-gauge locomotion and gregarious behavior. Cretaceous Research 23: 383–400.

[2] Mathews JC, Brusatte SL, Williams SA, Henderson MD (2009) The first *Triceratops* bonebed and its implications for gregarious behavior. Journal of Vertebrate Paleontology 29: 286–290.

[3] Rogers RR (1990) Taphonomy of three dinosaur bone bed in the upper Cretaceous Two Medicine Formation of northwestern Montana. Palaios 5: 394–413.

[4] Varricchio DJ, Horner JR (1993) Hadrosaurid and lambeosaurid bone beds from the Upper Cretaceous Two Medicine Formation of Montana: taphonomic and biologic implications. Canadian Journal of Earth Sciences 31: 997–1006.

[5] Zhao Q, Barrett PM, Eberth DA (2007) Social behaviour and mass mortality in the basal ceratopsian dinosaur *Psittacosaurus* (Early Cretaceous, People’s Republic of China). Palaeontology 50: 1023–1029.

[6] Salgado L, Canudo JI, Garrido AC, Carballido JI (2012) Evidence of gregariousness in Rebbachisaurids (Dinosauria, Sauropoda, Diplodocoidea) from the Early Cretaceous of Neuquén (Rayoso Formation) Patagonia, Argentina. Journal of Vertebrate Paleontology 32: 603–613.

[7] Barco JL, Canudo JI, Ruiz-Omeñaca JI (2006) New data on *Therangospodus oncalensis* from the Berriasian Fuentesalvo tracksite (Villar del Río, Soria, Spain): an example of gregarious behaviour in theropod dinosaurs. Ichnos 13: 237–248.

[8] Roach BT, Brinkman DL (2007) A reevaluation of cooperative pack hunting and gregariouness in *Deinonychus antirrhopus* and other nonavian theropod dinosaurs. Bulletin of the Peabody Museum of Natural History 48: 103–138.

[9] Colbert EH (1989) The Triassic dinosaur *Coelophysis* . Bulletin of the Museum of Northern Arizona 57: 1–160.

[10] Lockley MG, Matsukawa M (1999) Some observations on trackway evidence for gregarious behavior among bipedal small dinosaurs. Palaeogeography, Palaeoclimatology, Palaeoecology 150: 25–31.

[11] Moreno K, De Valais S, Blanco N, Tomlinson AJ, Jacay J, et al (2012) Large theropod footprint associations in western Gondwana: behavioural and palaeobiogeographic implications. Acta Palaeontologica Polonica 57: 73–83.

[12] Martínez RD, Novas FE (2006) *Aniksosaurus darwini* gen. et sp. nov., a new coelurosaurian theropod from the early Late Cretaceous of central Patagonia, Argentina. Revista del Museo Argentino de Ciencias Naturales 8: 243–259.

[13] Martínez RD, Giménez O, Rodríguez J, Luna M, Lamanna M (2004) An articulated specimen of the basal titanosaurian (Dinosauria: Sauropoda) *Epacthosaurus sciuttoi* from the early Late Cretaceous Bajo Barreal Formation of Chubut province, Argentina. Journal of Vertebrate Paleontology 24: 107–120.

[14] Chimsamy A, Raath MA (1992) Preparation of fossil bone for histological examination. Palaeontologia Africana 29: 39–44.

[15] Francillon-Vieillot H, de Buffrénil V, Castanet J, Géraudie J, Meunier FJ, et al.. (1990) Microstructure and mineralization of vertebrate skeletal tissues. 471–548. In: Carter JG ed. Skeletal biomineralization: patterns, processes and evolutionary trends. Volume 1. Van Nostrand Reinhold, New York. pp.832.

[16] Chinsamy Turan A (2005) The microstructure of dinosaur bone: deciphering biology with fine-scale techniques. The Johns Hopkins University Press. 195 p.

[17] Castanet J, Francillon-Vieillot H, Meunier FJ, de Ricqlès A (1993) Bone and individual aging. In Hall BK, ed. Bone. CRC, Boca Raton Press, Florida. pp.245–283.

[18] Ricqlès A, Meunier FJ, Castanet J, Francillon-Viellot H (1991) Comparative microstructure of bone. In Hall BK, ed. Bone matrix and bone specific products. CRC, Boca Raton Press, Florida. pp.1–78.

[19] Starck JM, Chinsamy A (2002) Bone microstructure and developmental plasticity in birds and others dinosaurs. Journal of Morphology 245: 232–246. 10.1002/jmor.10029 12386894

[20] Figari E, Hechem J, Homovc J (1990) Arquitectura depositacional de las areniscas verdes de la Formación Bajo Barreal, Provincia de Chubut. 3° Reunión Argentina de Sedimentología, San Juan, Argentina. Actas: 130–138.

[21] Novas FE, Ezcurra MD, Agnolin FL, Pol D, Ortíz R (2012) New Patagonian Cretaceous theropod sheds light about the early radiation of Coelurosauria. Revista del Museo Argentino de Ciencias Naturales 14: 57–81.

[22] Turner AH, Pol D, Clarke JA, Erickson GM, Norell MA (2007) A basal dromaeosaurid and size evolution preceding avian flight. Science 317: 1378–1381. 17823350 10.1126/science.1144066

[23] Carpenter K, Miles C, Cloward K (2005) New small theropod from the Upper Jurassic Morrison Formation of Wyoming. In Carpenter K, ed. The carnivorous dinosaurs. Bloomington Indiana University Press. pp.23–48.

[24] Seebacher F (2001) A new method to calculate allometric length-mass relationships of dinosaurs. Journal of Vertebrate Paleontology 21: 51–60.

[25] Hone DWE (2012) Variation in the tail lengh of non-avian dinosaurs. Journal of Vertebrate Paleontology 32: 1082–1089.

[26] Behrensmeyer AK (1978) Taphonomic and ecologic information from bone weathering. Paleobiology 4: 150–162.

[27] Kidwell SM, Fürsich FT, Aigner T (1986) Conceptual framework for the analysis and classification of fossil concentration. Palaios 1: 228–238.

[28] Hone DWE, Rauhut OWM (2010) Feeding behavior and bone utilization by theropod dinosaurs. Lethaia 43: 232–244.

[29] Kreutzer LA (1988) Megafaunal butchering al Lubbock Lake, Texas: a taphonomic reanalysis. Quaternary Research 30: 221–231.

[30] Behrensmeyer AK (1990) Terrestrial vertebrate accumulations In: Allison PA, Briggs DEG, eds. Taphonomy. Plenum Press. pp.291–335.

[31] Gutiérrez M, Kaufmann C (2007) Criteria for the identification of formation process in guanaco (*Lama guanicoe*) bone assemblages in fluvial-lacustrine environments. Journal of Taphonomy 5: 151–176.

[32] Johnson RG (1960) Models and methods for analysis of the mode of formation of fossil assemblages. Bulletin Geological Society of America 71: 1075–1085.

[33] Longrich NR, Horner JR, Erickson GM, Currie PJ (2010) Cannibalism in *Tyrannosaurus rex.* . Plos ONE 5: 1–6. 10.1371/journal.pone.0013419 PMC2955550 20976177

[34] Currie PJ, Trexler D, Koppelhus EB, Wicks K, Murphy N (2005) An unusual multi-individual tyrannosaurid bonebed in the Two Medicine Formation (Late Cretaceous, Campanian) of Montana (USA). In Carpenter K, ed. The carnivorous dinosaur. Bloomington Indiana University Press. pp.313–324.

[35] Raath MA (1990) Morphological variation in small theropods and its meaning in systematic: evidence from *Syntarsus rhodiensensis*. In Carpenter K, Currie PJ eds. Dinosaur systematics: approaches and perspectives. Cambridge University Press. pp.91–105.

[36] Eberth DA, Currie PJ (2010) Stratigraphy, sedimentology, and taphonomy of the *Albertosaurus* bonebed (upper Horseshoe Canyon Formation; Maastrichtian), southern Alberta, Canada. Canadian Journal of Earth Sciences 47: 1119–1143.

[37] Varricchio DJ, Sereno PC, Xijin Z, Lin T, Wilson JA, et al (2008) Mud-trapped herd captures evidence of distinctive dinosaur sociality. Acta Palaeontologica Polonica 53: 567–578.

[38] Coria RA, Currie PJ (2006) A new carcharodontosaurid (Dinosauria, Theropoda) from the Upper Cretaceous of Argentina. Geodiversitas 28: 71–118.

[39] Coria RA (2007) Nonavian theropods. In: Gasparini Z, Salgado L, Coria RA, eds. Patagonian Mesozoic reptiles. Bloomington & Indianapolis: Indiana University Press. pp.229–256.

[40] Berger J, Dulamseren S, Cain S, Enkkhbileg D, Litchman P, et al (2001) Back-casting sociality in extinct species: new perspectives using mass death assemblages and sex ratios. Proc. R. Soc. Lond. B. 268: 131–139. 10.1098/rspb.2000.1341 PMC1088582 11209882

[41] Isles TE (2009) The socio-sexual behaviour of extant archosaurs: implications for understanding dinosaur behavior. Historical Biology 21: 139–214.

[42] Molnar RE (2005) Sexual selection and sexual dimorphism in theropods. In Carpenter K, ed. The carnivorous dinosaur. Bloomington Indiana University Press. pp.284–324.

[43] Barden HE, Maidment SCR (2011) Evidence for sexual dimorphism in the stegosaurian dinosaur *Kentrosaurus aethiopicus* from the Upper Jurassic of Tanzania. Journal of Vertebrate Paleontology 31: 641–651.

[44] Carrano MT, Sampson SD, Forster CA (2002) The osteology of *Masiakasaurus knopfleri*, a small abelisauroid (Dinosauria: Theropoda) from the Late Cretaceous of Madagascar. Journal of Vertebrate Paleontology 22: 510–534.

[45] Kaliontzopoulou A, Carretero MA, Llorente GA (2007) Multivariate and geometric morphometrics in the analysis of sexual dimorphism variation in *Podarcis* lizards. Journal of Morphology 268: 152–165. 17236187 10.1002/jmor.10494

[46] Bonnan MF, Farlow JO, Masters SL (2008) Using linear and geometric morphometrics to detect intraspecific variability and sexual dimorphism in femoral shape in Alligator mississippiensis and its implications for sexing fossil archosaurs. Journal of Vertebrate Paleontology 28: 422–431.

